# Using Spinodal Decomposition to Investigate Diffusion Enhancement and Vacancy Population

**DOI:** 10.1002/advs.202412060

**Published:** 2025-02-18

**Authors:** Xinren Chen, Frédéric De Geuser, Alisson Kwiatkowski da Silva, Chuanlai Liu, Eric Woods, Dirk Ponge, Baptiste Gault, Dierk Raabe

**Affiliations:** ^1^ Max Planck Institute for Sustainable Materials 40237 Düsseldorf Germany; ^2^ CNRS University Grenoble Alpes Grenoble INP, SIMaP Grenoble F‐38000 France; ^3^ Department of Materials Royal School of Mines Imperial College London Prince Consort Road London SW7 2BP UK

**Keywords:** Al‐Zn alloy, diffusion enhancement, phase transition, spinodal decomposition, vacancy

## Abstract

Material sustainability requires energy‐efficient and rapid strengthening processes. In alloys, strengthening through diffusion‐driven precipitation is limited by the low vacancy concentration, with fewer than one vacancy per 100 billion lattice sites at room temperature in metals such as aluminum and iron under thermodynamic equilibrium. Artificially increasing vacancy concentrations by 1 to 7 orders of magnitude above equilibrium levels through quenching, irradiation, or deformation can significantly accelerate material strengthening. However, measuring vacancy concentrations below 10^−7^ in alloys and achieving spatial mapping remain challenging. Here, a vacancy‐mediated gradient microstructure near grain boundaries is reported and analyzed to investigate diffusion enhancement and the local vacancy population in an Al‐Zn system. This method uses cryogenic processes to preserve excess vacancies and halt microstructure evolution, enabling intermittent measurement of compositional fluctuations during ultrafast spinodal decomposition. It allows for the assessment of diffusion enhancement and determination of vacancy supersaturation in sub‐micrometer regions. Liquid nitrogen–quenched Al–12.5 at.% Zn alloy shows a vacancy concentration of ≈10^−7^ at room temperature, dropping to 10^−9^ after 3 h, with significant spatial variation near grain boundaries. This work addresses gaps in understanding the evolution and distribution of vacancies across various measurement scales, advancing the control of vacancies to enhance the strengthening of engineering alloys.

## Graphical abstract

Combining cryogenic sample preparation and intermittent atom probe tomography analyses enables the tracking of excess vacancy concentrations in as‐quenched Al‐Zn alloys. This approach reveals a vacancy‐mediated gradient spinodal decomposition microstructure near grain boundaries, with the ability to measure vacancy concentrations below 10^−7^. This study clarifies the spatial evolution of vacancies, advancing the control of vacancies to enhance the strengthening of materials.

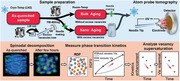

## 1 Introduction

In crystalline materials, a vacancy is a point defect characterized by an unoccupied position in the atomic lattice. The diffusion of substitutional elements proceeds through the exchange of positions with vacancies.^[^ ^1^ ^]^ The thermodynamic equilibrium site fraction of vacancies in aluminum is ≈10^−3^ near the melting point and decreases to below 10^−11^ at room temperature,^[^ ^2^, ^3^ ^]^ as determined by the Gibbs formation energy of vacancies that includes anharmonic contributions,^[^ ^4^ ^]^ leading to deviations from the common Arrhenius ansatz. In this work, the unit of vacancy concentration is consistently expressed as a site fraction. Despite their low concentration, vacancies are crucial for all diffusion‐controlled reactions, including ordering, precipitation, coarsening, dissolution, dislocation climb, and creep,^[^ ^5^, ^6^ ^]^ which are essential for controlling various mechanical and functional properties of materials.^[^ ^7^, ^8^ ^]^ Vacancy depletion near grain boundaries has been identified as a key factor in the formation of precipitate‐free zones,^[^ ^9^, ^10^ ^]^ which are negatively correlated with several interfacial decay phenomena, potentially leading to intergranular fractures.^[^ ^11^, ^12^ ^]^ Excess vacancies can be introduced to accelerate the aging strengthening processes in materials, as seen in methods such as irradiation,^[^ ^13^, ^14^ ^]^ cyclic heating and quenching (also known as “up‐quenching”)^[^ ^15^ ^],^ and deformation.^[^ ^16^ ^]^ These approaches can significantly reduce the energy and time required for the aging strengthening process.

Vacancies do not possess a physical entity like atoms, making them challenging to observe within materials.^[^ ^17^, ^18^, ^19^ ^]^ Quantifying vacancies typically relies on detecting changes in the crystal lattice, such as variations in lattice constants or changes in electrical resistance. However, these methods are only effective when the vacancy concentration is relatively high (>5 × 10^−5^).^[^ ^20^, ^21^ ^]^ The vacancy concentration can also be quantified through positron annihilation spectroscopy (PAS), with a probing depth of up to 0.5 millimeters,^[^ ^22^ ^]^ as vacancies induce changes in their electronic environment. PAS has been employed to measure vacancy concentration values as a function of time and thermal treatment.^[^ ^23^, ^24^ ^]^ Recent advancements have improved the spatial resolution of PAS—for instance, scanning positron microscopy can achieve beam sizes ≈1 µm,^[^ ^25^ ^]^ and a commercial SEM^[^ ^26^ ^]^ has been converted into a positron scanning microscope, enabling micron‐scale or even submicron‐scale analyses. Nevertheless, a detection limit for the vacancy concentration (>10⁻⁷)^[^ ^27^ ^]^ still exists for techniques such as PAS.^[^ ^28^, ^29^ ^]^ The fundamental challenges that lie in applying PAS include effects from precipitates that affect positron lifetimes, limited positron availability, and difficulties in preserving vacancy states after sample preparation,^[^ ^30^ ^]^ collectively hindering reliable submicrometer‐level vacancy distribution characterization. Consequently, key mechanisms related to the evolution and distribution of the vacancy population over time and temperature remain elusive.

To complement existing methods for quantifying vacancy populations across a wide measurement range, here we develop a new method that indirectly quantifies vacancy concentrations by measuring diffusion enhancement. The presence of excess vacancies significantly influences the kinetics of diffusion‐driven phase transitions and the resulting microstructural evolution. In turn, this provides an alternative pathway for quantifying vacancy populations through the detailed analysis and quantification of local microstructural changes arising from phase transitions during natural or artificial aging. Spinodal decomposition is one example of a time‐dependent and vacancy‐controlled process whose evolution can be monitored. Spinodal decomposition occurs when a single homogeneous supersaturated phase spontaneously separates into two (or more) precursor phases via “uphill diffusion”.^[^ ^31^, ^32^, ^33^ ^]^ Its phase transition kinetics can be analyzed by quantifying the composition fluctuations of solutes. The early stages of spinodal decomposition, and hence phase transition kinetics, can be studied through nanoscale microstructural and compositional analysis.^[^ ^34^ ^]^ Atom probe tomography (APT) can be systematically and quantitatively employed to track the progression of spinodal decomposition.^[^ ^35^, ^36^ ^]^ In a recent study of a model Al‐Zn alloy, we demonstrated how this can be used to study the room temperature diffusivity of Zn in Al.^[^ ^36^ ^]^

Here, by using cryogenically‐enabled workflows, we employ APT to analyze spinodal decomposition and thereby indirectly determine the distribution and evolution of the vacancy supersaturation from the earliest stages after quenching. The low temperature minimizes the substitutional diffusion of Zn atoms, providing ex‐situ observations of the microstructure evolution from the quench and over the course of natural aging, i.e., aging at room temperature. This includes observations over extended periods under conditions of high vacancy supersaturation and at varying distances from a vacancy sink, such as a surface or grain boundary. Our systematic study addresses longstanding questions about the distribution of quenched‐in vacancies in the alloy following quenching, and how the vacancy concentration decays during subsequent natural aging.

## 2 Result

### 2.1 Relationship Between Atomic Mobility and Vacancy Concentration

The diffusion in a solid solution is the net transport of species and is driven by a chemical potential gradient (∇μ). The net flux of solute atoms is described by *J*  =   −*MC*∇μ, where *M* and *C* are the atomic mobility and composition of the solute atoms, respectively. For substitutional diffusion, the atomic mobility *M* is proportional to the number of successful jumps an atom makes per second (Γ), where $\Gamma =zvX_{V}\exp\left(\frac{-\Delta G_{m}}{RT}\right)$. Here, *z* is the number of nearest neighbors, *v* the frequency of temperature‐independent atomic vibration, *X*_V_ the site fraction of vacancies in the lattice (also referred to as the vacancy concentration), Δ*G*_m_ the activation energy barrier for atomic migration, *R* is the gas constant, and *T* is the temperature. The vacancy concentration *X*_V_ directly impacts the number of available sites for atomic jumps, establishing the relationship that a higher vacancy concentration leads to greater atomic mobility.

The thermodynamic equilibrium vacancy concentration, $X_{V}^{e}$, is described by the equation $X_{V}^{e}=\exp\left(-\frac{\Delta G_{V}}{RT}\right)$, where Δ*G*_V_ is the molar formation free energy of vacancies. When a sample is quenched from a high temperature, the system can temporarily retain the vacancies that existed at that higher temperature, leading to vacancy supersaturation. The degree of supersaturation is defined as the ratio between the actual vacancy concentration and the new equilibrium vacancy concentration ($X_{V}/X_{V}^{e}$).

### 2.2 Phase Transition Kinetics as a Probe of Vacancy Supersaturation

**Figure 1:**
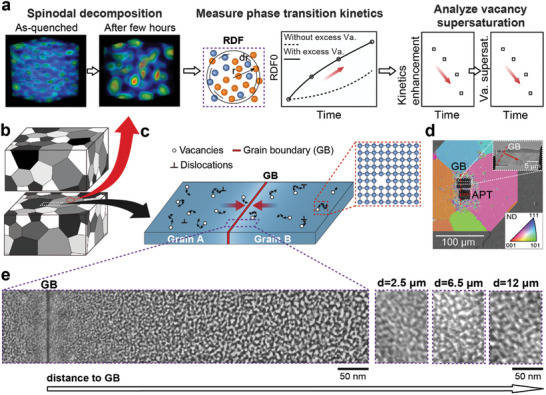
Workflow of probing vacancy supersaturation by phase transition kinetics. a) Illustration of the workflow for measuring local phase transition kinetics enhancement and vacancy supersaturation in a submicron‐sized region within the bulk material. b) Illustration of the sampling positions inside a sliced bulk sample for quantifying local phase transition kinetics and vacancy supersaturation (red arrow) and spatial difference around a grain boundary (black arrow). c) Illustration of the diffusion and annihilation of excess vacancies to vacancy sinks. d) Scanning‐electron micrograph that illustrates the location of TEM sample preparation and APT specimens adjacent to a grain boundary (GB), with overlaid, the inverse pole figure from electron‐backscatter diffraction (EBSD) providing the out‐of‐plane grain orientation. Inset is an overview of the TEM lamella. e) High‐angle annular dark‐field (HAADF) image of the bulk naturally aged Al‐12.5 at.% Zn alloy near the grain boundary and at longer distances from the grain boundary with “d” representing the distance to the grain boundary.

In **Figure** **1**a,b, we illustrate the principle of assessing local vacancy supersaturation within a grain by measuring the enhancement of phase transition kinetics. In regions with vacancy supersaturation, the atomic mobility *M* is positively correlated with the degree of vacancy supersaturation. Consequently, the spinodal decomposition can be significantly accelerated by the magnitude of the vacancy supersaturation, while neglecting the influence of vacancies on the chemical driving force, as described by the Cahn–Hilliard equation:^[^ ^37^ ^]^

(1) $$\frac{\partial C}{\partial t}=\nabla \cdot \left(M\nabla \frac{\delta F}{\delta C}\right)$$

where *t* is the time, and *F* is the free energy of the system.

For a micro‐sized region inside a quenched bulk experiencing rapid spinodal decomposition, after aging for a while, the region can be extracted from the sliced bulk, as shown in Figure 1b. The state of spinodal decomposition can then be effectively assessed by comparing it to the process without excess vacancies. This can be achieved by calculating and processing radial distribution functions (RDFs) using APT, as suggested in refs. [35, 36] An RDF represents the probability of finding an atom of a given species at a distance “*r*” from a reference atom, averaged over each atom within a region of tens of nanometers of a given dataset.^[^ ^38^ ^]^ By comparing the evolution of the RDFs in systems with and without vacancy supersaturation, we can determine the level of vacancy supersaturation, as shown in Figure 1a. We applied this protocol to uncover the distribution and evolution of vacancies in an Al‐12.5 at.% Zn alloy after quenching from 793 K. As Al‐12.5 at.% Zn lies within the spinodal decomposition region^[^ ^39^ ^]^ and avoids excessively high Zn concentrations that would induce premature decomposition during cooling, this alloy composition offers an optimal balance of driving force and stability for spinodal decomposition, enabling more accurate theoretical and experimental analysis.

A key challenge in the study of vacancies is that they annihilate fast at structural defects. In polycrystalline materials, grain boundaries are microstructural imperfections characterized by a higher free volume than the bulk, and they contain intrinsic defects such as grain boundary dislocations. Thus, after quenching, grain boundaries act as effective vacancy sinks, absorbing and annihilating excess vacancies,^[^ ^40^, ^41^ ^]^ which results in the formation of a low vacancy concentration zone in their vicinity, as demonstrated in Figure 1c. However, the width of the zone with low vacancy concentration and the evolution of vacancy supersaturation within these zones have not been thoroughly characterized. Differences in local vacancy concentration should lead to spatial variations in the local diffusion process of substitutional atoms. Consequently, regions nearer to grain boundaries are expected to exhibit a less advanced state of spinodal decomposition. This is confirmed by scanning transmission electron microscopy (STEM) performed on a lamella prepared near a grain boundary with a misorientation of 18°, as shown in Figure 1d,e, after natural aging for 1 day. Figure 1e shows a progressively coarser arrangement of compositional fluctuations with increasing distance from the grain boundary, indicating a more advanced state of spinodal decomposition further from the grain boundary.

**Figure 2:**
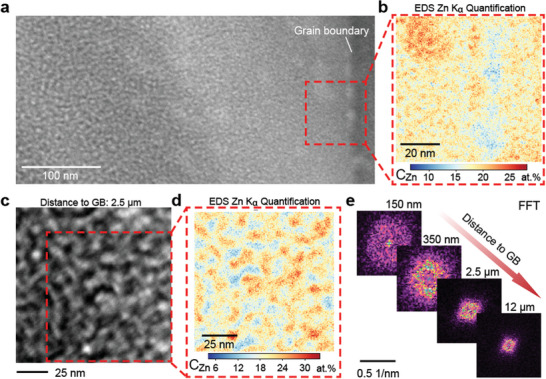
Gradient microstructure of spinodal decomposition near a grain boundary in Al‐Zn alloys. a) High‐angle annular dark‐field (HAADF) image of the one‐day bulk naturally aged Al‐12.5 at.% Zn alloy near the grain boundary. b) EDS Zn Kα quantification map from the boxed region in (a), showing compositional fluctuations in Zn concentration. c) HAADF image taken 2.5 µm away from the grain boundary, illustrating spinodal decomposition features. d) Corresponding EDS Zn Kα quantification map from (c), revealing local Zn concentration variations. e) FFT (Fast Fourier Transform) analysis of regions at increasing distances (150 nm, 350 nm, 2.5 µm, and 12 µm) from the grain boundary, highlighting the evolution of compositional wavelengths and coarsening of spinodal decomposition.

**Figure** **2** illustrates the EDS (Energy‐Dispersive X‐ray Spectroscopy) results of the gradient microstructure of spinodal decomposition near a grain boundary in the Al‐12.5 at.% Zn alloy after one day of natural aging. In the HAADF image (Figure 2a), the region near the grain boundary shows a fine, heterogeneous microstructure, indicative of spinodal decomposition. The EDS Zn K_α_ quantification map (Figure 2b) reveals compositional fluctuations in Zn concentration, highlighting the local solute clustering. Moving further into the grain interior, the STEM image taken 2.5 µm away from the grain boundary (Figure 2c) displays more developed spinodal decomposition features. This is supported by the corresponding EDS Zn K_α_ map (Figure 2d), which shows localized variations in Zn content. The FFT (Fast Fourier Transform) analyses (Figure 2e) of regions at increasing distances from the grain boundary (150 nm, 350 nm, 2.5 µm, and 12 µm) demonstrate the evolution of the spinodal decomposition wavelength and the coarsening of compositional fluctuations with increasing distance. These observations provide clear evidence of a vacancy‐mediated gradient microstructure near grain boundaries, influencing the progression of spinodal decomposition within the alloy. While analysis of STEM images using methods such as 2D FFT can provide insights into wavelength and coarseness, it cannot fully capture 3D compositional variations or accurately quantify the amplitude of compositional fluctuations—a key parameter reflecting the progress of spinodal decomposition. Local EDS mapping results, as shown in Figure 2b,d, can offer qualitative insights into elemental distributions, however, its chemical resolution is still limited. Therefore, we conducted APT experiments, for near‐atomic scale, 3D compositional measurements, enabling a more comprehensive and quantitative characterization of spinodal decomposition.

### 2.3 The Spatial Variation in Diffusion Enhancement

**Figure 3:**
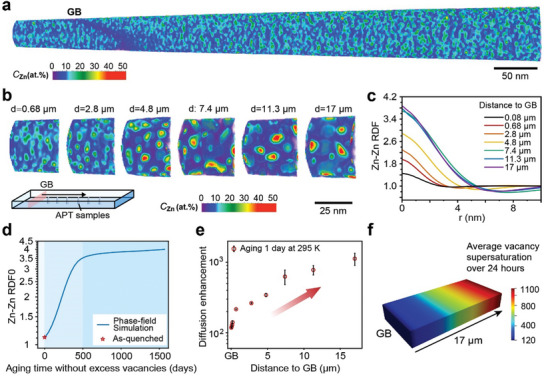
Mapping long‐range excess vacancy distribution. a) Volume Zn composition mapping of the APT results near a grain boundary of the bulk naturally aged Al‐12.5 at.% Zn alloy after 1 day. b) APT volume Zn composition mapping of a series of samples that were bulk naturally aged for 1 day, with perpendicular distances to the grain boundary ranging from 0.68 to 17 µm. c) Radial distribution functions (RDFs) of APT result at increasing distances from the grain boundary. The data point at 0 nm is obtained by extrapolating the curve from the adjacent 0.2 nm data point. d) Zn‐Zn RDF0 curve from phase‐field simulation of spinodal decomposition of Al‐12.5 at.% Zn alloy at 295 K without excess vacancies. e) Diffusion enhancement results for samples at increasing distances from the grain boundary. f) Mapping of average vacancy supersaturation over 24 h, from the region close to the grain boundary to a distance of 17 µm. The color bar shows a dimensionless value representing the ratio between the average supersaturated vacancy concentration and the equilibrium vacancy concentration.

To quantify the phase transition kinetics, we employed APT to analyze samples from the region near the grain boundary to the interior of the grain (Figures S1,S2, Supporting Information). The inherently 3D nature of APT data facilitates the detailed quantification of composition fluctuations within the samples. The results shown in **Figure** **3**a,b demonstrate that as the distance from the grain boundary increases, the microstructure resulting from spinodal decomposition becomes coarser. To exclude the influence of Zn depletion around grain boundaries^[^ ^42^ ^]^ as grain boundary precipitates consume nearby solute atoms, the local average composition was studied. The results show that the zone with a lower Zn composition compared to other regions is limited to only 50 nm from the grain boundary (Figure S3, Supporting Information). Thus, Figure 3a,b suggest that spinodal decomposition occurs more rapidly further away from the grain boundaries.

To statistically analyze composition fluctuation, radial distribution functions (RDFs) were calculated. Figure 3c displays the Zn‐Zn RDF curves. The analysis of the RDFs enables us to track and quantify the progression of spinodal decomposition, as discussed in ref. [35] Extrapolation of the RDF curves toward *r* = 0 (RDF0) indicates the amplitude of the mean square of the composition fluctuation (see Experimental Section). As shown in Figure 3c, the RDF0 value increases as the distance to the grain boundary increases from 0.08 to 7.4 µm. This analysis confirms that regions further from the grain boundary, extending into the grain interior, exhibit faster spinodal decomposition. However, at distances beyond 7.4 µm, the RDF curves remain nearly unchanged, indicating that vacancy supersaturation remains within the same range, as does the advancement of spinodal decomposition. This suggests a critical distance from the grain boundary, beyond which vacancy supersaturation is significantly less affected by grain boundaries.

The rise of RDF0 can be studied using phase‐field modeling,^[^ ^36^ ^]^ which simulates the evolution of the composition field without excess vacancies, from which RDF0 can be calculated (see Experimental Section). During spinodal decomposition, RDF0 increases, as plotted in Figure 3d, making it a good indicator of the phase transition state. To establish a reference starting point, the RDF0 value at 8 µm from the bulk sample surface in the as‐quenched sample—obtained using cryogenically‐enabled workflows (described in more detail in the Section 2.4)—was experimentally determined and superimposed in Figure 3d.

Using this reference value of RDF0, provided by the blue curve in Figure 3d, the measured values of RDF0 in Figure 3c are used to determine an equivalent aging time for cases without excess vacancies required to reach this state of spinodal decomposition. The ratio of the equivalent aging time to the actual natural aging time provides insights into the diffusion enhancement, as depicted in Figure 3e. It should be noted that these values reveal the average diffusion enhancement over the course of the day rather than the transient diffusion enhancement at the time of measurement.

As mentioned earlier, diffusion enhancement is a direct effect of vacancy supersaturation. Consequently, the diffusion enhancement data have been further converted to a corresponding map of the average vacancy supersaturation over 24 h, as shown in Figure 3f. For instance, at a distance of 17 µm from the grain boundary, after quenching from 793 K, the average vacancy supersaturation within a day can reach ≈1000. In contrast, at distances less than 200 nm from the grain boundary, the vacancy supersaturation is only ≈100. This difference can be attributed to the absorption of excess vacancies by the grain boundary, resulting in lower vacancy supersaturation near the grain boundary.

### 2.4 Local Evolution of the Spinodal Decomposition

**Figure 4:**
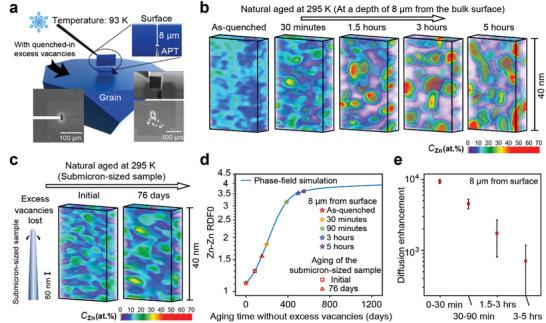
Evolution of local vacancy supersaturation inside a grain. a) Illustration of ex‐situ sample preparation using a cryogenic stage to temporarily freeze microstructure evolution in focused‐ion‐beam (FIB) equipment. b) Evolution of Zn volume composition mapping at a depth of 8 µm from the surface during natural aging at 295 K, quenched from 793 K by liquid nitrogen. c) Zn volume composition mapping in a submicron‐sized sample during natural aging at 295 K. d) Summary of RDF0 at various time points at a depth of 8 µm from the surface. The curve represents the simulated Zn–Zn RDF0 during the spinodal decomposition of the Al‐12.5 at.% Zn alloy at 295 K without excess vacancies. e) Evolution of the average diffusion enhancement, specifically at a depth of 8 µm from the surface.

To probe the very early stages of decomposition far from vacancy sinks, we introduce a cryogenic workflow that significantly slows the phase transition during sample preparation, allowing for ex‐situ observation of the microstructure evolution during the first 5 h of natural aging post‐quenching, as shown in **Figures** **4**a and S4 (Supporting Information). Following quenching into liquid nitrogen, the sample was stored and transported into the focused ion beam (FIB) at near liquid nitrogen temperature (93 K). At this temperature, the Zn interdiffusion coefficient is 10^16^ times lower than at 295 K, given the same vacancy concentration. (see Figure S5, Supporting Information). This cryogenic procedure can temporarily “freeze” the microstructure changes, even in the presence of a high supersaturation of vacancies.

Using a recently introduced cryogenic specimen preparation protocol^[^ ^43^ ^]^ (Figure S6, Supporting Information), APT specimens were prepared ≈8 µm below a pre‐polished surface (Figure 4a), which is considered a sink that annihilates excess vacancies. Specimens were prepared at least 50 µm away from other grain boundaries to minimize their influence on microstructural evolution. After the preparation of a set of specimens at ≈93 K, the stage was heated to 295 K inside the FIB to allow natural aging to proceed for 30 min to 5 h (Figure S4, Supporting Information). Subsequently, the sample was cooled again to ≈93 K to freeze the microstructure evolution and prepare a new set of APT specimens.

The results in Figure 4b demonstrate the evolution of spinodal decomposition over time for this region. To compare this with spinodal decomposition without excess vacancies, we prepared a submicron‐sized Al‐12.5 at.% Zn sample, in which excess vacancies are difficult to retain and quickly annihilate at the sample's surface due to the size effect.^[^ ^30^ ^]^ Thus, spinodal decomposition in this sample is considered to occur without excess vacancies. Figure 4c displays the spinodal decomposition in the submicron‐sized sample. The spinodal decomposition state observed during the first APT experiment is referred to as the initial state. The APT experiment was then conducted on the sample again after natural aging at 295 K for 76 days.

To determine the fictitious equivalent aging time at equilibrium vacancy concentration for the samples aged under excess vacancy conditions, the points are placed on the blue line in Figure 4d representing the phase‐field calculation according to their measured RDF(0) values. The initial state shown in Figure 4c corresponds to an aging time of 83 days without excess vacancies from the as‐quenched state obtained using the cryogenic workflow. This is due to the spinodal decomposition occurring with excess vacancies during sample preparation. The APT result after natural aging at 295 K for 76 days (from the initial state), as shown in Figure 4c, corresponds to an aging time of 153 days without excess vacancies from the as‐quenched state. The spinodal decomposition microstructure shows a similarity between the result after just 30 min of natural aging in the bulk sample with excess vacancies and the result after 76 days of natural aging in the submicron‐sized sample without excess vacancies. The increase in the spinodal decomposition state in the bulk sample (located 8 µm below the surface) after just 30 min of aging, as represented by the required aging time without excess vacancies, is greater than the increase in the spinodal decomposition state in the submicron‐sized sample between the initial state and after 76 days of aging.

The diffusion enhancement from this series of ex‐situ experiments is plotted as a function of the effective aging period in Figure 4e. Within 30 min after the quench, the estimated diffusion enhancement at a depth of 8 µm below the surface can reach up to 9445 ± 622 times that of the case without excess vacancies. This value then drops to 4560 ± 720 between 30 min and 1.5 h, further decreases to 1746 ± 936 between 1.5 and 3 h, and eventually falls to 706 ± 660 between 3 and 5 h. The results suggest that vacancy supersaturation experiences a significant decrease during natural aging.

### 2.5 The Diffusion Enhancement Near Grain Boundaries

**Figure 5:**
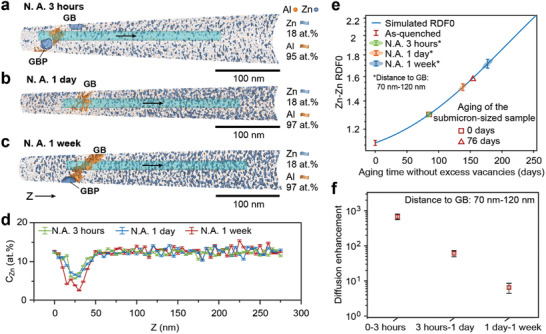
Vacancy supersaturation near grain boundaries. Atom probe tomography results of a) Bulk naturally aged (N. A.) for 3 h sample. b) Bulk naturally aged for 1‐day sample. c) Bulk naturally aged for 1‐week sample. d) 1D regionally averaged composition mappings in the Z‐direction of Zn within cylinders (Φ15 nm) shown in a–c), using a step size of 5 nm. e) Determination of aging states using RDF0 values at different natural aging durations following quenching from 793 K, in the region 70–120 nm from the grain boundary. The curve represents the simulated Zn–Zn RDF0 during the spinodal decomposition of the Al‐12.5 at.%Zn alloy at 295 K without excess vacancies. f) Diffusion enhancement over time in the region 70–120 nm from the grain boundary. “Distance” represents the perpendicular distance to the grain boundary.

After analyzing the diffusion enhancement evolution in the interior of the grain, our attention shifted to the region near the grain boundary, where the diffusion enhancement can differ significantly from that in the grain interior. A series of APT specimens near grain boundaries were prepared to further investigate vacancy supersaturation in these regions. Each specimen was subjected to progressively longer durations of natural aging. All grain boundaries had a similar misorientation of ≈11° and showed no low‐number coincident‐site‐lattice (CSL) (Σ < 50) relationships, as shown in Figures S3,S7,S8 (Supporting Information). **Figure** **5**a–c display reconstructed APT datasets for bulk materials subjected to natural aging for 3 h, 1 day, and 1 week. Orange isosurfaces (95–97 at.% Al) delineate the location of the grain boundary, while teal isosurfaces highlight regions enriched in Zn (C_Zn_ > 18 at.%), indicating composition fluctuations (see also Figure S9, Supporting Information).

It should be noted that the Zn composition around grain boundaries can be lower than in the grain center, as grain boundary precipitates (GBP) consume nearby Zn atoms.^[^ ^10^ ^]^ In Figure 5d, composition profiles calculated along cylindrical regions of interest (Φ15 nm) at 5 nm intervals reveal a widening of the low Zn composition region, with a width of ≈50 nm

The Zn–Zn RDF0 data from regions situated 70–120 nm from the grain boundary are plotted in Figure 5e, revealing an increasing trend in RDF0 from as‐quenched state to 1 week. For comparison, the increase in RDF0 during the aging process in the submicron‐sized sample of the same composition over 76 days is significantly lower than the increase of RDF0 from 3 h to 1 week, indicating the existence of vacancy supersaturation in this region. As plotted in Figure 5f, within the initial 3‐h period after quenching, the average diffusion enhancement can reach up to 680±123. It then drops to 61±12 from 3 h to 1 day and 6±2 after 1 day to 1 week. Our findings reveal that vacancy supersaturation, in the region 70–120 nm away from grain boundaries, persists after 1 day of aging.

## 3 Discussion

We have introduced an approach to examining vacancy supersaturation by characterizing the advancement of spinodal decomposition in a binary Al‐12.5 at.% Zn alloy. This measurement is crucial for understanding the relationship between quenching conditions, subsequent natural aging, and the resulting vacancy distribution. However, some features require further discussion.

As shown in Figures 1, 2, 3, the state of spinodal decomposition can be quantified by both the STEM and the APT method. This offers indirect, qualitative access to the assessment of vacancy concentrations. This is done by examining the local spinodal decomposition microstructure, using EDS or APT probing, providing information about the compositional fluctuations. These composition changes over time can then be related to metrics such as atomic mobility and the Landau free energy landscape from which the driving forces derive. While these features do not directly yield vacancy concentrations, the underlying enhanced atomic mobility values can be used as a metric to infer the corresponding higher vacancy concentrations, i.e., the degree of vacancy supersaturation. Although STEM can cover larger areas and provide a broader overview of spinodal decomposition evolution, it is generally limited to 2D projections.

In contrast, APT provides nanoscale, 3D compositional mapping, allowing for more precise and quantitative characterization of spinodal decomposition features. Both STEM and APT have their strengths: STEM is well‐suited for rapid, qualitative assessments over larger areas, whereas APT excels at providing detailed, 3D insights at the nanoscale. By combining these complementary techniques, we can establish a more robust foundation for interpreting the state of spinodal decomposition. This integrated approach could, in the future, provide a more direct link between STEM observations and the APT‐based RDF analysis, thereby strengthening the validity of methods for estimating vacancy supersaturation.

The method is based on the indirect relationship between local phase transition kinetics and vacancy concentration. Although it cannot directly provide the vacancy concentration, it is used as a qualitative metric for the local vacancy supersaturation.

Figure 3 connects the observed local diffusion enhancement to vacancy supersaturation. By presenting the data in this form, we confirmed the existence of a vacancy concentration gradient near grain boundaries, a phenomenon proposed theoretically for decades.^[^ ^10^ ^]^ The results also show that the inhomogeneous phase transition process near the grain boundaries extends over several micrometers, rather than being restricted to a few hundred nanometers as commonly reported for precipitate‐free zones.^[^ ^10^, ^44^, ^45^ ^]^

Such findings reveal that, at the early stages of aging, vacancy‐mediated solute clustering as precursors to precipitates can exhibit spatial inconsistencies. This inconsistency influences subsequent artificial aging by affecting local precipitate nucleation rates within grains. These insights highlight that grain boundaries act as efficient vacancy sinks, enabling long‐distance interactions with the evolving microstructure. This understanding is essential for developing strategies to control and optimize the early‐stage aging behavior of Al alloys.

The local evolution observation of vacancy supersaturation in the bulk of a grain, probed here 8 µm below the surface and tens of microns away from any grain boundary as summarized in Figure 4, we measured an average diffusion enhancement reaching 10^4^ within the first 30 min post‐quenching. This suggests that the vacancy concentration in the interior of the grains maintains a high vacancy concentration (equivalent to ≈10^−7^), which is 10^4^ times the predicted equilibrium vacancy concentration of ≈10^−11^ at 295 K in Al. We show that this high vacancy concentration induces rapid spinodal decomposition in the sample, leading to the development of a mature spinodal decomposition stage after 5 h. At this stage, further investigation of spinodal decomposition is not recommended, as changes in the RDF become minimal and measurement errors increase substantially.

By comparing the ratio of equilibrium vacancy concentration in Al between 793 and 295 K, which is ≈10^7^, we observed that the vacancy concentration experiences a substantial drop during quenching and the early stages of aging. Several factors could contribute to this drop. First, the annihilation of excess vacancies at surfaces and grain boundaries significantly reduces the vacancy concentration. Second, the trapping of vacancies by solute atoms^7^ and solute clusters decreases the number of mobile vacancies that contribute to diffusion.^[^ ^46^, ^47^ ^]^ Finally, the high supersaturation of vacancies promotes the formation of bivacancies, trivacancies, and vacancy clusters,^[^ ^48^ ^]^ which further inhibit diffusion enhancement.

Since Zn exhibits a lower solute–vacancy binding energy compared to other solutes, such as Sn, Pb, and In,^[^ ^49^ ^]^ the solute‐trapping effect in Al–Zn alloys is only a moderate feature at the start of the aging process. As demonstrated in Figure 4, even after 5 h, a significant diffusion enhancement still exists. However, as precipitates grow or undergo phase transitions, solute trapping becomes more pronounced. This increased trapping effect may introduce greater uncertainty in indirect vacancy concentration measurements because diffusivity is primarily determined by free (untrapped) vacancies. As the trapping effect intensifies, it alters the effective diffusivity, potentially leading to inconsistencies in vacancy‐related measurements.

Additionally, in regions near the grain boundary (70–120 nm away), we measured a vacancy concentration of 10^−8^ within 3 h after quenching, which then dropped to a level of 10^−9^–10^−10^ after a total of 3 h. This reduction is attributed to the grain boundaries acting as vacancy sinks.^[^ ^10^ ^]^ Notably, even in these near grain boundary regions, diffusion remains significantly enhanced —≈100–1000 times faster than under vacancy equilibrium conditions—during the first day of natural aging. These findings refine our understanding of precipitation behavior in the precipitate‐free zones. By revealing vacancy concentration levels in these areas, our results provide new insights into controlling the formation of precipitate‐free zones around grain boundaries, as observed in aging‐hardened alloys such as Al–Cu,^[^ ^10^ ^]^ Al–Zn,^[^ ^50^ ^]^ Al–Li^[^ ^51^ ^]^ alloys, and others.

## 4 Conclusion and Outlook

This study reveals the presence of a gradient microstructure in terms of the spinodal decomposition near grain boundaries. By leveraging the quantification of the kinetics of spinodal decomposition, we establish an effective and indirect method to probe local vacancy supersaturation. By using accelerated diffusion as a proxy for measuring vacancies, our approach circumvents the challenges associated with solute‐vacancy interactions that often complicate the use of conventional techniques—such as positron‐annihilation spectroscopy or resistivity measurements—particularly in the presence of solute precipitation. Although we focused on an Al‐12.5 at.% Zn alloy, the underlying principle can be applied to a wide range of spinodal decomposition‐strengthened alloys, such as Fe‐Cr,^[^ ^35^ ^]^ and Mg‐Li‐Al.^[^ ^52^ ^]^ Since the method relies on the connection between the kinetics of diffusion‐driven phase transitions and vacancy supersaturation, other diffusion‐driven phase transitions can also act as effective indicators, such as the growth of nanoparticles during precipitation^[^ ^53^ ^]^ and chemical ordering processes.^[^ ^54^ ^]^ Consequently, this approach could broaden the application scope and enhance our ability to detect vacancy supersaturation near structural defects, improving both detection limits and spatial resolution.

By quantifying the spinodal decomposition kinetics accelerated by excess vacancies, we have revealed a substantial vacancy supersaturation—on the order of 10^3^–10^4^ times above the equilibrium vacancy concentration—persisting within the grain for the first 1.5 h of aging at 295 K following liquid‐nitrogen quenching. After 3 h, this supersaturation decreased by roughly one order of magnitude. Additionally, we mapped the spatial variation of vacancy supersaturation from regions near the grain boundary (70–120 nm away) up to 17 µm into the grain interior. The average vacancy supersaturation over 24 h rose from ≈10^2^–10^3^. Near the grain boundary, it dropped from 10 to 6 after one day, corresponding to a qualitative vacancy concentration level of ≈10^−9^–10^−11^. These measurements reach vacancy concentration levels far below the current detection limits of other methods, demonstrating the potency of using this new approach for probing extremely low vacancy concentrations qualitatively.

These findings provide new insights into how vacancies are distributed and evolve within quenched materials, demonstrating the profound influence of vacancy supersaturation on early‐stage phase transformations. We show thatthe vacancy concentration gradient can extend well into the grain interior due to grain boundaries acting as vacancy sinks, resulting in a long‐range inhomogeneity of the phase transition process during the early stages of natural aging. By measuring local diffusion enhancements, we detect a vacancy concentration gradient that evolves over significant distances. Our approach thus lays the groundwork for future investigations into the distribution and temporal evolution of excess vacancies induced by quenching, deformation, or irradiation, offering a more comprehensive understanding of vacancy‐mediated phenomena in materials science.

## 5 Experimental Section

### Materials

The materials utilized in this study (Al‐12.5 at.% Zn) were initially synthesized in a vacuum induction furnace and cast as a rectangular billet using high‐purity metals. The as‐cast ingots underwent homogenization at 793 K. Subsequently, samples were prepared with dimensions of 4 mm × 4 mm × 9 mm and solutionized at 793 K for 2 h before quenching. For samples used to study ex situ microstructure evolution below the surface, liquid nitrogen was employed to quench the sample, thereby immediately freezing the microstructure evolution and inhibiting the diffusion of vacancies after quenching. The other samples were quenched by high‐speed helium gas flow, followed by storage at 295 K for natural aging. Both high‐speed helium gas flow and liquid nitrogen provided quenching rates ranging from −150 to −50 K s^−1^ from 793 to 295 K.

### FIB Preparations

Atom probe tomography specimens were prepared and mounted on silicon micro‐tip coupons using a Xe source FEI Helios focused‐ion beam (PFIB) instrument equipped with a cryogenic sample stage with built‐in thermal couples and heaters, and a cryogenic easy‐lift manipulator. This setup was capable of cooling down from 295 to 253 K within 5 min, to 93 K within 30 min, and heating from 93 to 273 K in 20 min. For the sample quenched with liquid nitrogen, the sample was transferred to the PFIB sample stage under cryogenic temperatures. Cryo‐lift‐out and cryo‐welding procedures^[^ ^55^ ^]^ were used (Figure S6, Supporting Information) to mount the APT specimen and conducted sharpening at 93 K. After sharpening, the connection of APT specimens to the silicon post was reinforced with Pt redeposition at room temperature. For samples aged for 1 day and 1 week, the APT specimens were prepared by FIB lift‐out and sharpening at room temperature.

### Microstructure Observations

The crystal orientation mapping was performed using electron backscatter diffraction (EBSD) on a ZEISS Sigma instrument. For transmission electron microscopy (TEM), specimens were prepared through in‐situ lift‐out and thinning, utilizing the Xe source FEI Helios plasma focused ion beam (PFIB). The electron transmission microstructures of samples were analyzed using a JEOL‐2200FS instrument operated at 200 kV.

### APT Experiments

The APT data acquisitions were conducted using a Cameca Instrument Inc. Local Electrode Atom Probe (LEAP) 5000XR. The samples were operated in voltage mode with a pulse fraction of 15%, and a frequency of 200 kHz at a temperature of 70 K. Reconstruction and analyses were carried out using the commercial program IVAS in AP Suite 6.1. The reconstruction parameters, including the field factor (*Kf*) and image compression factor (*ICF*), were calibrated based on the observed angles between the chosen poles and the crystalline interplanar spacing of the selected poles.^[^ ^56^ ^]^ The mapping of Zn composition in the volume was performed using AP Suite 6.1, employing a grid size of 0.5 nm and a delocalization parameter set to 3 nm. For datasets containing grain boundaries, the presence of the grain boundaries was assessed by observing changes in the pole figures on the detector hitmap (see Figures S3,S7,S8, Supporting Information).

### Phase‐Field Simulations

The evolution of spinodal decomposition was simulated via the Cahn–Hilliard equation (Equation (1)).^[^ ^37^, ^57^ ^]^ Simulations were carried out by using public libraries for fast Fourier transform in MATLAB (Mathworks Inc.). The CALPHAD‐based Gibbs energy of the substitutional phase was used in the phase‐field simulation and was based on the assessment of the Al‐Zn system.^[^ ^58^ ^]^ The atomic mobility data and interfacial gradient coefficient were provided by previous work,^[^ ^36^ ^]^ which thoroughly evaluated the spinodal decomposition of the Al‐12.5 at.% Zn alloy at 295 K. The RDF0 evolution of the system could be calculated as follows:^[^ ^35^ ^]^

(2) $$RDF0=\frac{1}{V}\;\int \int \int \;{\left(\frac{C_{Zn}\left(x,y,z\right)}{C_{0}}\right)}^{2}dxdydz$$

where *C_Zn_* is the local Zn composition, *C*_0_ the bulk average Zn composition and *V* is the volume of the system. When a sinusoidal composition fluctuation, $C_{Zn}\;\left(x,y,z\right)=C_{0}+A\sin\left(\frac{2\pi }{\lambda }\left(x+y+z\right)\right)$ (as described by Cahn),^[^ ^31^ ^]^ is substituted into Equation (2), it yields *RDF*0 = 1  +  *A* ^2^2*C*_0_^2^.Thus, the amplitude of composition fluctuation during spinodal decomposition can be determined by $2A=2C_{0}\sqrt{2(RDF0-\;1)}$. The value of *RDF*0 is therefore a critical index that reflects the progress of spinodal decomposition.

## Conflict of Interest

The authors declare no conflict of interest.

## Supporting information



Supporting Information

## Data Availability

The data that support the findings of this study are available from the corresponding author upon reasonable request.
