# Supplementary material for: Using Spinodal Decomposition to Investigate Diffusion Enhancement and Vacancy Population
Source: Adv Sci (Weinh). 2025 Feb 18;12(14):2412060. doi: 10.1002/advs.202412060 (PMC11984890; doi:10.1002/advs.202412060)
Supplement: Supplementary file 1 — Supporting Information [file ADVS-12-2412060-s001.docx]

**Supporting information**

**Using Spinodal Decomposition to Quantify Diffusion Enhancement and Vacancy Population**

**Xinren Chen, Frédéric De Geuser, Alisson Kwiatkowski da Silva, Chuanlai Liu, Eric Woods, Dirk Ponge, Baptiste Gault, Dierk Raabe**

This PDF file includes:

Figures S1 to S9

**
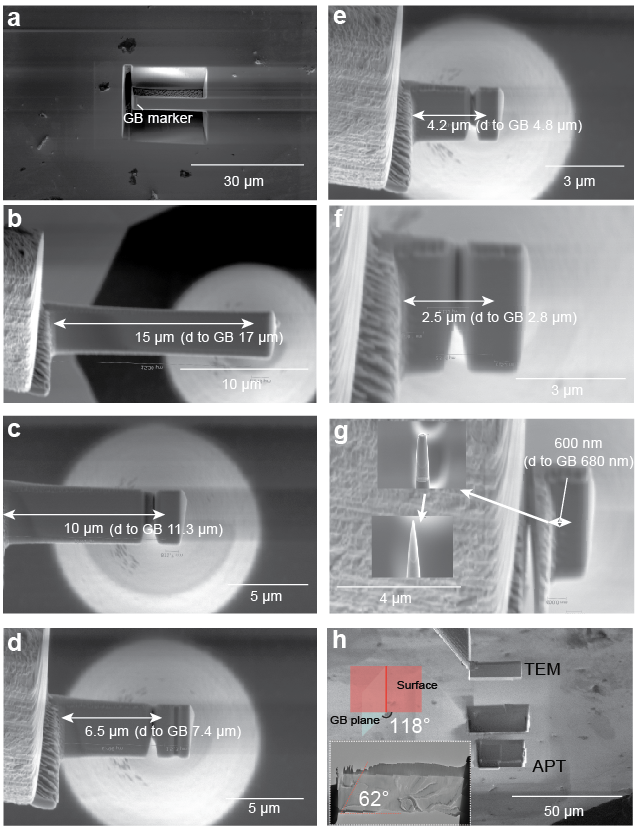
**

**Figure S1.** a-g. Preparing samples with different distances to the grain boundary. h. Preparation of TEM sample to determine the angle between the surface and grain boundary plane.

**
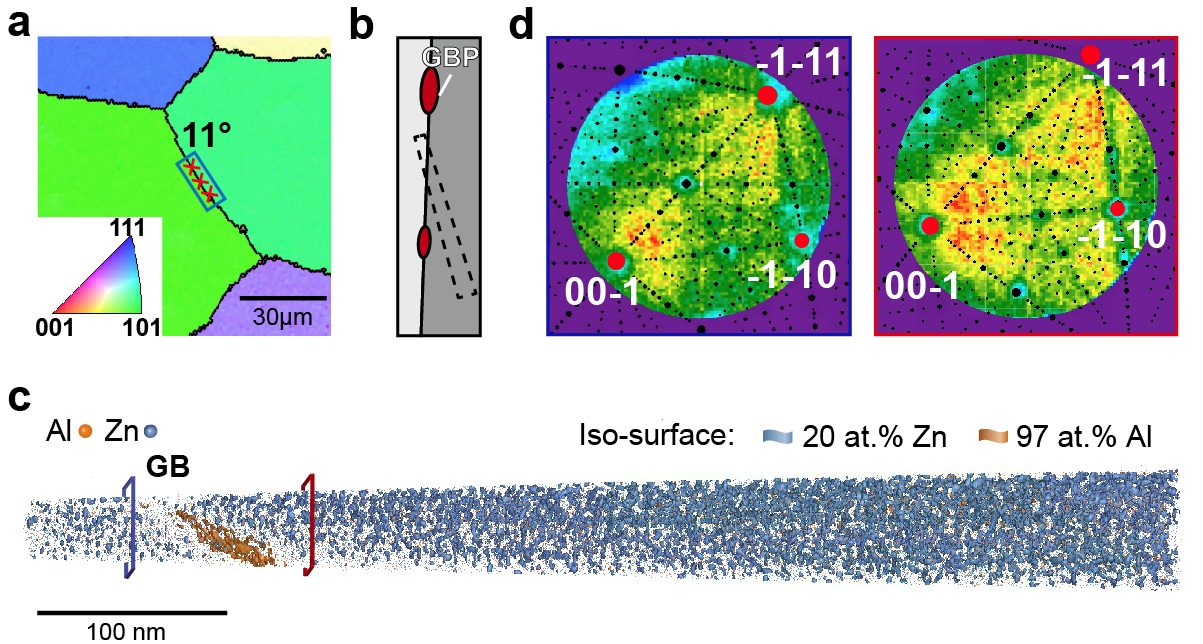
**

**Figure S2.** a. Electron backscatter diffraction mapping (EBSD) of the bulk naturally aged (N.A.) for 1 day sample. The blue box delineates the region near the grain boundary that was extracted to make the needles. b. Illustration depicting the location of the needle at a grain boundary. c. Iso-surfaces highlighting the spinodal decomposition microstructure near the grain boundary of N.A. for 1 day sample. d. Detector maps of the regions marked by the blue and red frames in (c) show differences in pole figures and orientation, which are used to confirm the location of the grain boundary.


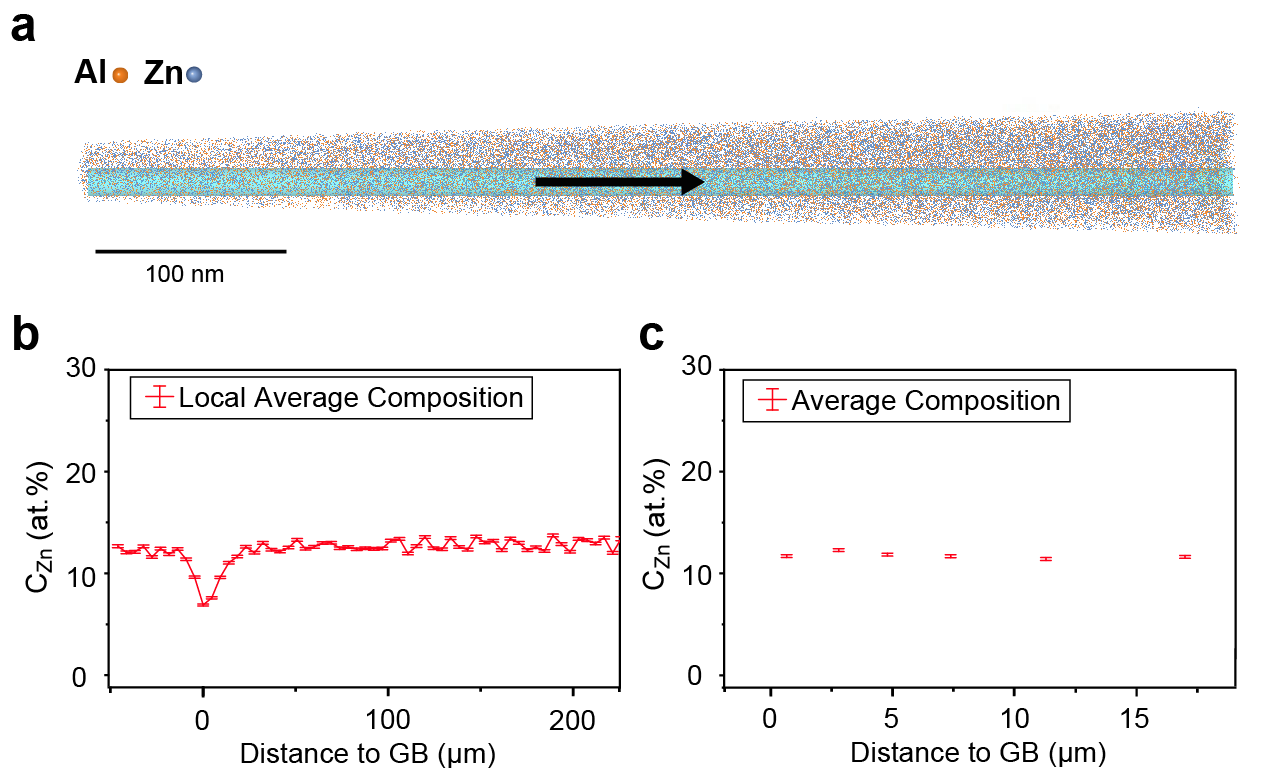


**Figure S3.** a. The cylinder region (Φ:15 nm) in the naturally aged (1 day) sample shown in Fig. 3a. b. The average composition of Zn within the cylinder through the APT needle sample, with a step size of 5 nm. c. The average composition of the series of samples shown in Fig. 3b, with perpendicular distances to the grain boundary ranging from 0.68 μm to 17 μm.

**
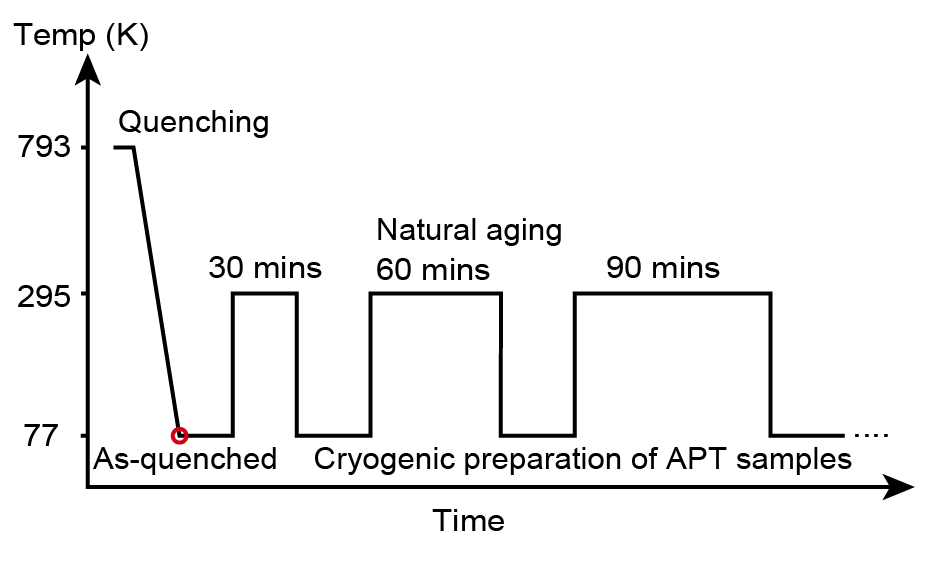
**

**Figure S4.** The quenching, aging, cryogenic procedures, and temperature cycles involved in the quasi-in-situ sample preparation were used to analyze the evolution of spinodal decomposition at a constant distance of 8 μm from the surface of the sliced grain.


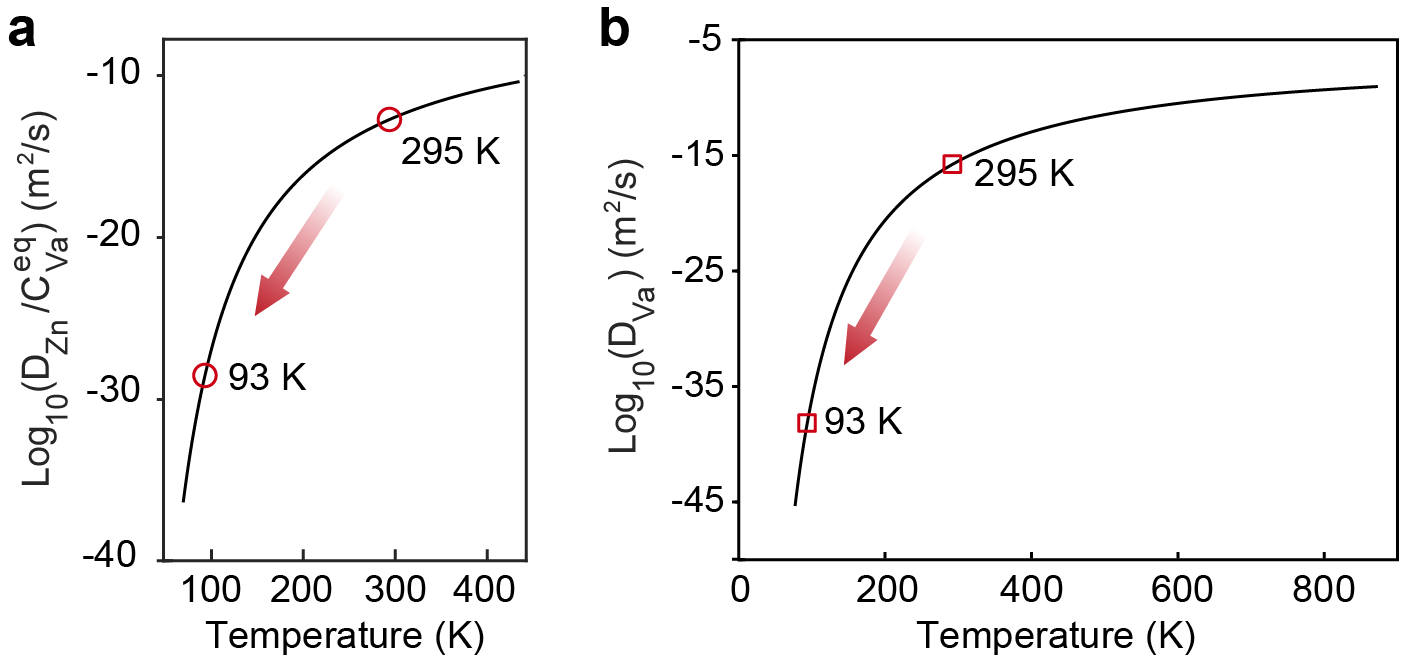


**Figure S5.** a. The specific Zn diffusivity, calculated by dividing the Zn interdiffusion coefficient in Al-12.5 at.% Zn (obtained from the diffusivity database^1^) by the calculated equilibrium vacancy concentration in Al using the vacancy formation energy from the work^2^ at each temperature. This reflects the variation in Zn interdiffusion coefficient across different temperatures, while maintaining a constant vacancy concentration. b. The calculated diffusion coefficient of vacancies in pure aluminum, as determined using the diffusivity database^1^. At 93 K, the diffusion coefficient of vacancies is10^22^ times lower than at 295 K, significantly slowing the diffusion and annihilation of vacancies.


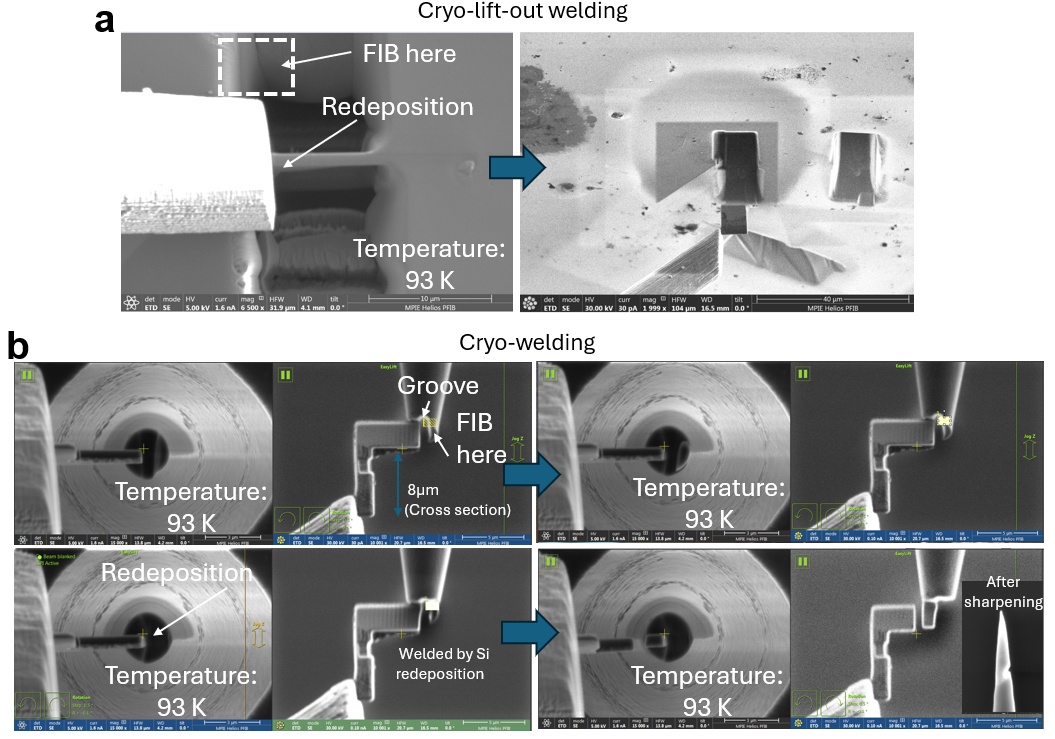


**Figure S6.** Illustration of cryo-lift-out (a) and cryo-welding (b) procedures used in this work. To perform cryo-lift-out and cryo-welding, the sample and FIB stage are maintained at cryogenic temperatures (around 93 K) to prevent damage and limit diffusion during processing. Using the FIB, a trench is created around the area of interest while minimizing damage to the surrounding material. The micromanipulator is then carefully positioned to touch the edge of the lamella, followed by FIB milling in a designated region, which causes redeposition of material between the lamella and the micromanipulator without the need for a gas injection system to deposit a metal. Afterward, the cryo-welded sample is lifted out using the micromanipulator and positioned onto a Si post. FIB milling is then performed on a portion of the Si post to induce Si redeposition between the lamella and the post, effectively welding the sample in place. Finally, the sample is refined by additional FIB milling. All processes are conducted at cryogenic temperatures to prevent rapid diffusion in the sample.

**
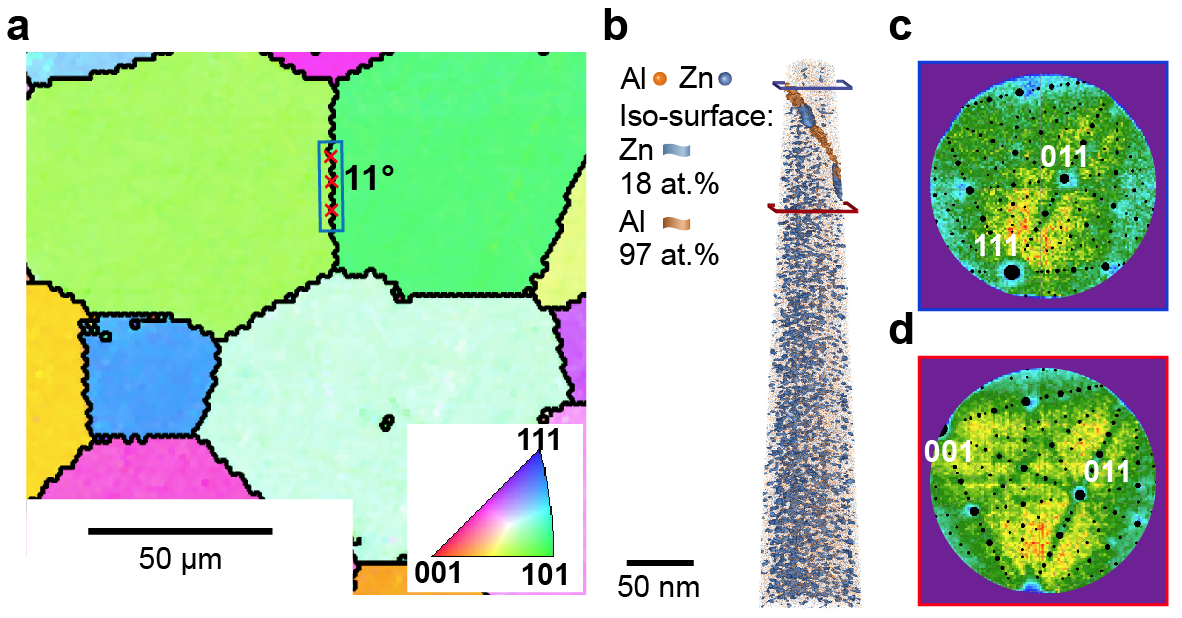
**

**Figure S7.** a. Electron backscatter diffraction (EBSD) mapping of the Al-12.5 at.% Zn sample, bulk naturally aged (N.A.) for 3 hours. The blue box highlights the region near the grain boundary that was extracted to prepare the APT needles. b. Iso-surfaces highlighting the spinodal decomposition microstructure near a grain boundary in the Al-12.5 at.% Zn sample, bulk naturally aged at room temperature for 3 hours. c. Detector map of the region marked by the blue frame in b. d. Detector map of the region marked by the red frame in b, showing differences in the pole figure compared to c, which is used to confirm the location of the grain boundary.

**
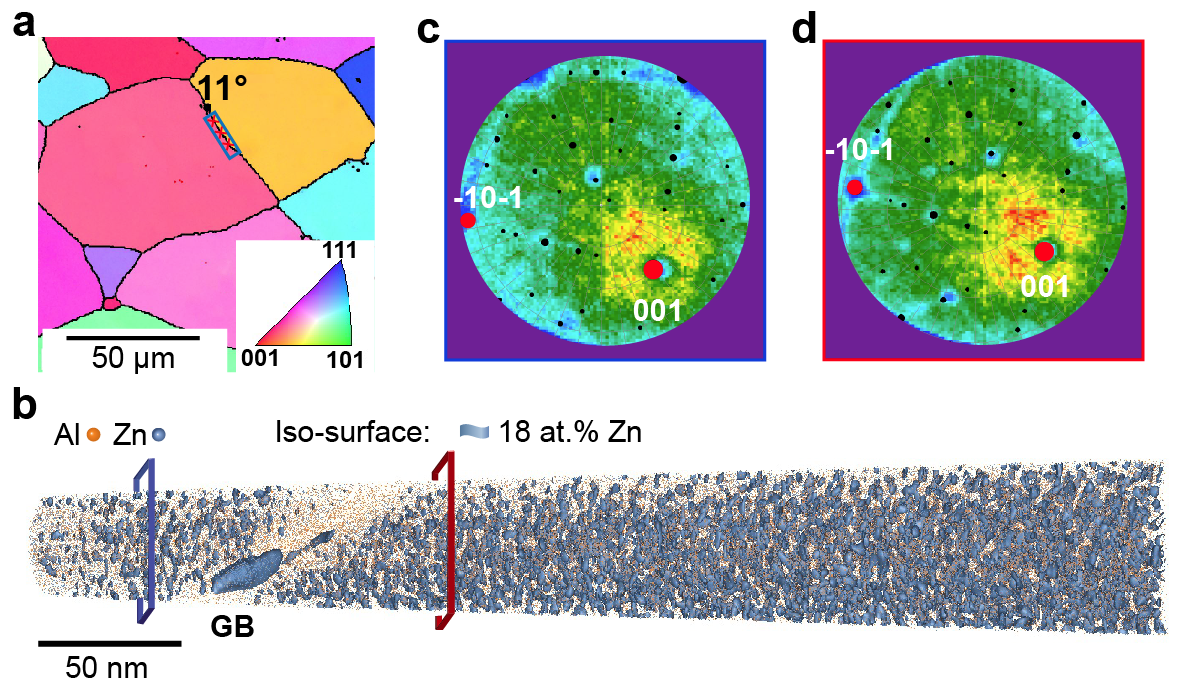
**

**Figure S8**. a. Electron backscatter diffraction (EBSD) mapping of the Al-12.5 at.% Zn sample, bulk naturally aged (N.A.) for 1 week. The blue box indicates the region near the grain boundary that was extracted to prepare the APT needles. b. Iso-surfaces highlighting the spinodal decomposition microstructure near the grain boundary in the sample bulk naturally aged for 1 week. c. Detector map corresponding to the region marked by the blue box in b. d. Detector map of the region marked by the red frame in b, showing differences in pole figures compared to c, which is used to confirm the location of the grain boundary.

**
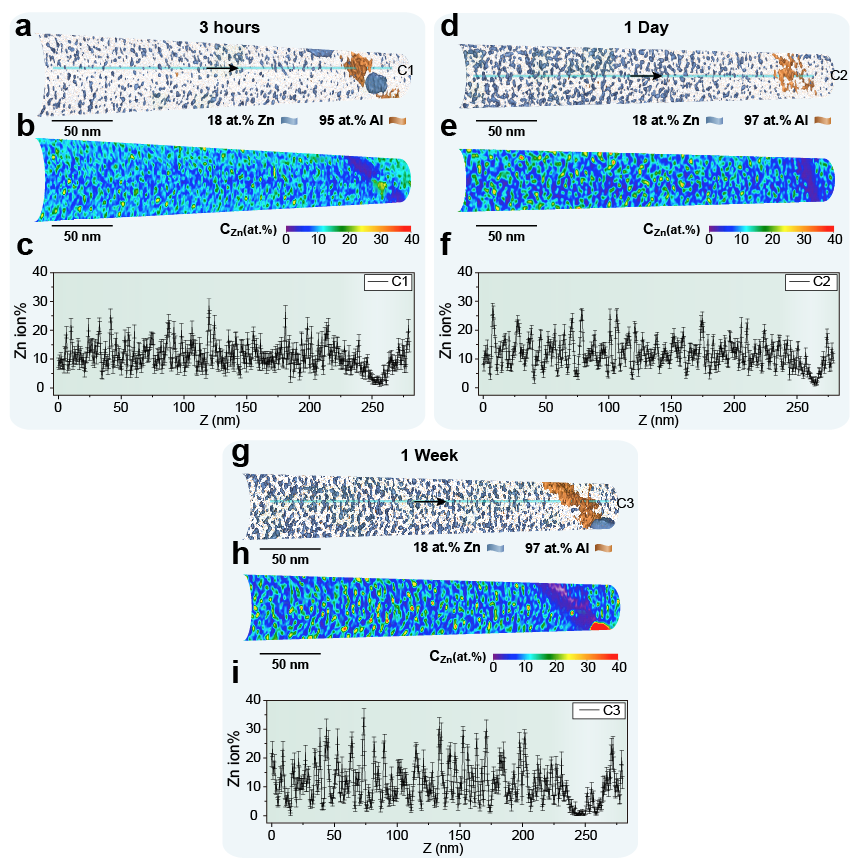
**

**Figure S9:** Bulk naturally aged for 3 hours sample results: a. Slice of atom probe tomography dataset with isosurfaces. b. Volume composition mapping of Zn (voxel size: 0.5 nm × 0.5 nm × 0.5 nm). c. One-dimensional composition mapping of Zn in the Z direction for the cylinder in a. Bulk naturally aged for 1 day sample results: d. Slice of atom probe tomography dataset with isosurfaces. e. Volume composition mapping of Zn (voxel size: 0.5 nm × 0.5 nm × 0.5 nm). f. One-dimensional composition mapping of Zn in the Z direction for the cylinder in d. Bulk naturally aged for 1 week sample results: g. Slice of atom probe tomography dataset with isosurfaces. h. Volume composition mapping of Zn (voxel size: 0.5 nm × 0.5 nm × 0.5 nm). i. One-dimensional composition mapping of Zn in the Z direction for the cylinder in g.

**References:**

1. Cui, Y.W., Oikawa, K., Kainuma, R. & Ishida, K. Study of diffusion mobility of Al−Zn solid solution. *J. Phase Equilib. Diffus.* **27**, 333-342 (2006).

2. Glensk, A., Grabowski, B., Hickel, T. & Neugebauer, J. Breakdown of the Arrhenius Law in Describing Vacancy Formation Energies: The Importance of Local Anharmonicity Revealed by Ab initio Thermodynamics. *Physical Review X* **4**, 011018 (2014).
